# Nanoantibiotics Based in Mesoporous Silica Nanoparticles: New Formulations for Bacterial Infection Treatment

**DOI:** 10.3390/pharmaceutics13122033

**Published:** 2021-11-29

**Authors:** Elena Álvarez, Blanca González, Daniel Lozano, Antonio L. Doadrio, Montserrat Colilla, Isabel Izquierdo-Barba

**Affiliations:** 1 Departamento de Química en Ciencias Farmacéuticas, Universidad Complutense de Madrid, Instituto de Investigación Sanitaria, Hospital 12 de Octubre i+12, Plaza Ramón y Cajal s/n, 28040 Madrid, Spain; elealvar@ucm.es (E.Á.); blancaortiz@ucm.es (B.G.); danlozan@ucm.es (D.L.); antoniov@ucm.es (A.L.D.); 2 CIBER de Bioingeniería, Biomateriales y Nanomedicina, CIBER-BBN, 28040 Madrid, Spain

**Keywords:** nanoantibiotics, mesoporous silica nanoparticles, targeted therapies, stimuli-response, infection treatment, metal ions

## Abstract

This review focuses on the design of mesoporous silica nanoparticles for infection treatment. Written within a general context of contributions in the field, this manuscript highlights the major scientific achievements accomplished by professor Vallet-Regí’s research group in the field of silica-based mesoporous materials for drug delivery. The aim is to bring out her pivotal role on the envisage of a new era of nanoantibiotics by using a deep knowledge on mesoporous materials as drug delivery systems and by applying cutting-edge technologies to design and engineer advanced nanoweapons to fight infection. This review has been divided in two main sections: the first part overviews the influence of the textural and chemical properties of silica-based mesoporous materials on the loading and release of antibiotic molecules, depending on the host–guest interactions. Furthermore, this section also remarks on the potential of molecular modelling in the design and comprehension of the performance of these release systems. The second part describes the more recent advances in the use of mesoporous silica nanoparticles as versatile nanoplatforms for the development of novel targeted and stimuli-responsive antimicrobial nanoformulations for future application in personalized infection therapies.

## 1. Introduction

Infections associated with bone implants are one of the most severe and overwhelming threats to society today [1,2]. The implant surface constitutes a perfect environment for bacterial adhesion, growth and colonization [3]. In addition, the wear produced by the micromotions of these bone prostheses provokes the release of residues that elicit inflammation local, which provides an ideal place for the appearance of infections [4]. Nowadays, although significant improvements in prophylaxis and aseptic surgical procedures have remarkably decreased the prevalence of implant infections, the infection rates remain in the 1–2% range [5]. Moreover, the reinfection rate following revision surgical procedures are also extremely elevated (ca. 33%) [6], which increases the expenses associated with the treatment. These bone implant infections are caused by pathogens such as *Staphylococcus aureus* [7], whose resistance mechanisms make it resilient and invincible [8]. Among the difficulties of eradicating implant-associated infections, biofilm formation is the most challenging and outstanding one, since it provides the bacteria with a perfect environment for longstanding survival [9,10]. The action of antibiotics is prevented by these biofilms, which serve as an impenetrable physical barrier [11]. In addition, the bacteria existing into the biofilm are especially pathogenic due to their phenotypic diversity, which confers resistance to antimicrobial treatment, making the dose of drugs to be administered up to 1000 times greater than that necessary for their planktonic phenotype [9,12]. Another major concern is that biofilm causes irreversible damage, provoking bone resorption, due to both the direct bacteria’s intrinsic aggressiveness and the indirect associated inflammatory processes [13,14].

Once an infection is detected in an orthopedic prosthesis, the major hurdle is its complete elimination from the affected area, including the implant and the adjacent necrotic regions. Usually, antibiotics are massively administered to the patient during long-time hospitalization periods. Unfortunately, most of the cases require a second surgical procedure to replace the infected prosthesis at the same time that a local treatment using antibiotic loaded poly(methylmethacrylate) beads is applied [15]. Nonetheless, the total eradication of the infected areas is a difficult task, and latent bacteria are responsible for recurrent and antimicrobial resistant infections. The high morbidity and patient suffering, as well as the associated economic burdens for the national healthcare systems, have motivated the scientific community to devote much research effort to the development of alternative therapies able to circumvent the limitations of those used nowadays [16]. Currently, there is not any consensual and efficient therapy for the management of bone implant infection; there is an urgent need to find a solution to this changeling problem [15,17].

New therapeutic advancements should be aimed at avoiding the need of implant removal and replacement by means of the in situ treatment of the infected region, in a localized manner and with the greatest possible specificity. Nanotechnology has entered into this arena, providing powerful tools to design and engineer nanoparticles as nanoweapons to fight bacterial infection much more effectively than conventional antimicrobial treatments. These nanoparticles are envisioned as targeted nanomedicines for local treatments, achieving higher antimicrobial effect at low doses, and therefore reducing toxicity and side effects. Among nanoparticles, there are those with inherent antimicrobial properties, such as metal (Ag, Au, etc.) and metal oxide (ZnO, CuO, TiO_2_, etc.) nanoparticles [18,19,20] and nanoparticles acting as nanocarriers of antimicrobial agents, the so-called nanoantibiotics [21,22].

Among nanocarriers, mesoporous silica nanoparticles (MSNs) are one of the most promising ones due to their interesting structural and textural properties, which allow them to host a wide range of different therapeutic cargoes for drug delivery in different biomedical applications, including cancer, osteoporosis and infection, among others [23,24,25,26,27,28,29,30,31,32,33,34,35].

**Figure 1:**
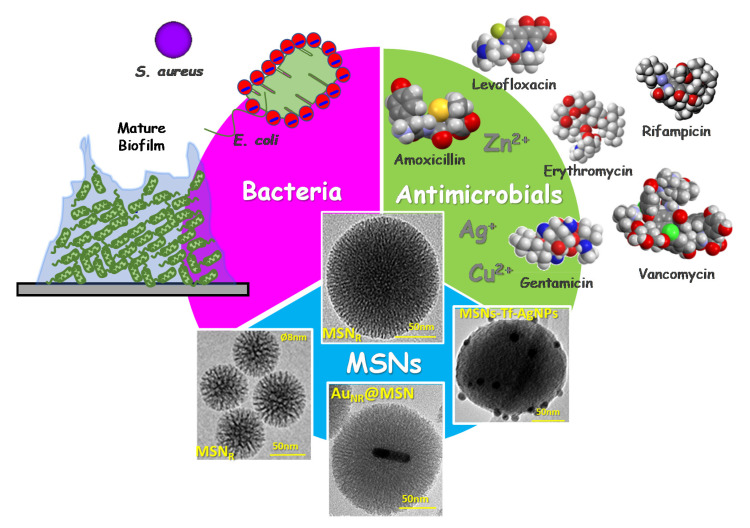
Schematic depiction of the three main actors starring in the innovative scientific approaches developed by professor Vallet-Regí’s research group in the design and engineering of nanoantibiotics for infection treatment: bacteria (in planktonic state or biofilms), antimicrobial agents (including antibiotics alone or in combination with metal ions) and mesoporous silica nanoparticles (MSNs). MSN_R_: Radial mesoporous silica nanoparticles; Au_NR_@MSN: gold nanorods@mesoporous silica nanoparticles; MSN-Tf-AgNPs: mesoporous silica nanoparticles decorated with transferrin and silver nanoparticles.

In the field of bone infection, professor Vallet-Regí and co-workers envisaged the design and development of advanced nanoantibiotics as a challenging scientific scenario, starring three main actors: bacteria, antimicrobials and MSNs (Figure 1). Firstly, bacteria, which are present in bone implant infection, either in planktonic state or forming highly resistant biofilms, must be eradicated in situ in a localized fashion, to avoid the need of surgical procedures to remove and replace the infected prosthesis. On the other hand, antimicrobials (including antibiotics alone or in combination with metal ions) should be locally delivered at the infected target site at small doses with high specificity, which would increase the antimicrobial efficacy and reduce the risk of harmful side effects in healthy organs, tissues and cells. This goal can be accomplished by using nanocarriers able to load, protect and transport antimicrobials to the target (bacteria and/or biofilm). Once there, the nanocarriers steadily release antimicrobials in response to certain internal or external stimuli. Among nanocarriers, MSNs emerged as the third actor that fulfils all of these requirements. Thus, innovative cutting-edge technologies are being applied to design and engineer innovative targeted stimuli-responsive antimicrobials delivery MSNs-based nanosystems.

This review aims to provide a comprehensive overview of the most recent scientific advances carried out by professor Vallet-Regí’s research group in the field of MSNs for bacterial infection treatment [36]. First, the role of silica-based mesoporous materials as drug delivery devices of antimicrobial agents is described. The influence of the textural properties (pore diameter, surface area and pore volume), chemical nature of the surface of these materials and host–guest interactions, on the loading and release kinetics of different antibiotics is revised. Furthermore, the potential of molecular modelling in the design of these controlled delivery devices and a deep comprehension on their performance as release systems of antibiotics of different families is also described. Finally, the more recent scientific approaches aimed to design and develop advanced nanoantibiotics for future applications in personalized therapies are tackled.

## 2. Engineering Mesoporous Materials as Antimicrobial Delivery Systems

**Figure 2:**
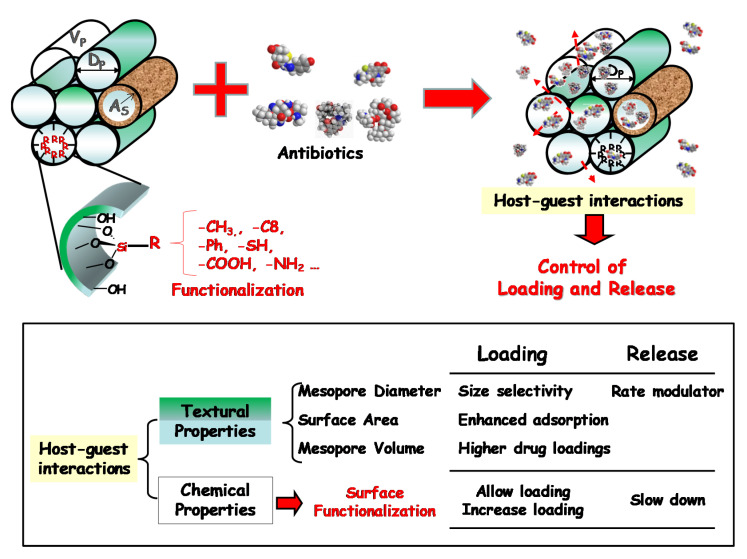
Influence of the textural and chemical properties of the silica-based mesoporous matrices in their performance as drug delivery systems.

The pioneering research on MCM-41 material as a controlled delivery system of ibuprofen [37], an anti-inflammatory used as a model drug, inspired the design and engineering of an important number of silica-based mesoporous matrices to host diverse antimicrobial agents such as antibiotics [26,28,29,30,38,39,40,41]. The reasons that account for the high impact of mesoporous materials in the field of controlled drug delivery rely on their distinctive structural (ordered pore structure), textural (narrow pore size distributions, large surface areas and high surface volumes) and chemical (high density of silanol groups that allows the covalent grafting of organic groups) properties, which are pivotal factors in the performance of these systems during the loading and release of drug molecules (Figure 2) [31,42,43,44].

The pore diameter restricts the maximum size that the drug molecules can have to enter the channels of the mesoporous material, therefore being a size-selectivity parameter during the drug loading process. This parameter also limits the diffusion of the cargo molecules to the delivery medium, therefore acting as a release rate modulator. The loading of molecules into the mesopores depend on the adsorptive capacity of the host and consequently on the surface area of the matrix. Thus, large surface areas in the host increase the contact time with the guest molecule, which results in higher amounts of loaded drug. Finally, the pore volume may increase the amount of loaded drug if pore filling is accomplished, which involves an increase in the drug–drug interactions inside the mesoporous cavities [42,43].

With respect to the chemical properties, the amorphous silica surface is covered by siloxane bridges and silanol groups. The magnitude of the silanol number, that is, the number of OH groups per unit of surface area, is considered to be a physico-chemical constant when the surface is hydroxylated to the maximum degree. This constant has the numerical value of 4.9 OH/nm^2^ (arithmetical mean) and is known in the literature as the Kiselev–Zhuravlev constant [45,46]. These silanol groups provide many opportunities to modulate the host–guest interactions [47]. Thus, when pure silica mesoporous matrices are used, their interaction with the drug molecules would take place through weak interactions, such as Van der Waals forces or hydrogen bonds. Nevertheless, it is feasible to functionalize such silanol-containing mesoporous groups by covalently anchoring diverse organic groups, which leads to a full family of organic–inorganic hybrid materials [42,48,49,50]. The two main approaches used to functionalize silica mesoporous matrices are the post-synthesis method, also known as silanization or grafting, and the one-pot synthesis or co-condensation route. The post-synthesis method is generally carried out under anhydrous conditions by reaction of organosilanes of the (R’O)_3_SiR type, with the free silanol groups of the host matrix. This is a versatile method that allows the selective functionalization of either the external surface of the mesopore, when the process is performed before the surfactant removal, or the entire silica surface, i.e., the inner and external mesoporous surface, when the procedure is carried out once the surfactant has been extracted. Such functionalization provokes a decrease in the textural properties of the resulting material, due to presence of organic moieties into the mesopore voids, which is accompanied by an increase in the wall thickness. On the other hand, the co-condensation route is a one-step method comprising the simultaneous hydrolysis and condensation reactions of both silica and organosilica precursors in the presence of a surfactant as a structure-directing agent. In this last method, there is a more homogeneous distribution of the organic functions covering the entire silica surface, but there is an upper functionalization threshold above to avoid the disorder of the mesoporous structure [48].

Functionalization of mesoporous matrices permits a precise modulation of drug loading and release kinetics [43], as a result of the different host–guest interactions via electrostatic attractive forces, hydrophilic–hydrophobic interactions or electronic interactions [47,51,52].

**Figure 3:**
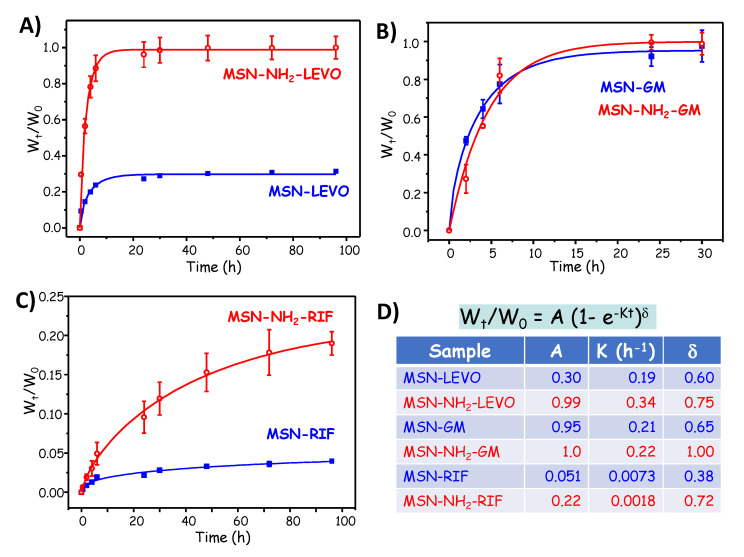
Drug release curves of three antibiotics: (**A**) levofloxacin (LEVO); (**B**) gentamicin (GM); and (**C**) rifampicin (RIF), from pure-silica (MSN) and amino-modified (MSN-NH_2_) MSNs. (**D**) Kinetic parameters derived from the fit of the experimental data to theoretical kinetic model using the equation W_t_/W_0_ = A (1 − e^−kt^)^δ^, where W_t_ is the amount of antibiotic released at t time, W_0_ is the initial amount of loaded drug, A is the maximum antibiotic release at infinite time, k is the release kinetic constant and δ is a dimensionless non-ideality parameter. Adapted with permission from Aguilar et al. [53], Micropor. Mesopor. Mat., published by Elsevier, 2021.

Figure 3 shows the drug release curves of three antibiotics of different chemical nature, namely levofloxacin (LEVO), gentamicin (GM) and rifampicin (RIF) from pure-silica (MSN) and amino-modified (MSN-NH_2_) MSNs [53]. In such research work, MSN-NH_2_ was used as a model nanoplatform, since amino-functionalization of mesoporous matrices are widely reported in the literature [44]. The different release profiles observed in each case account for the different nature of the host–guest interactions, and also to the chemical properties of the antibiotic molecule. For comparative purposes, the experimental data were fitted to a typical diffusion first order release kinetics model [54]. In the case of LEVO, there is an initial burst effect followed by a more sustained release; moreover, there is a partial antibiotic retention in the pure silica MSN (Figure 3A). This behavior is in good agreement with the strong attracting interactions via hydrogen bonds of LEVO molecules and the silanol groups present in bare silica MSN, as previously reported [55,56,57]. On the contrary, the repulsive interactions of the antibiotic molecule with protonated amino groups in the host matrix at physiological conditions would trigger the total antibiotic release from LEVO loaded in MSN-NH_2_. Concerning GM, despite the different existing host–guest interactions, the high solubility of this molecule is the predominant factor that provokes the total antibiotic release from both nanomaterials with similar profiles (Figure 3B). Finally, in the case of RIF, the lack of burst effect and noticeable drug retention can be mainly explained by the low solubility and relatively high molecular size of such an antibiotic [58].

The above described research study reveals that the release of antibiotics of different families from mesoporous matrices strongly depends both on the chemical properties of the drug itself and the host–guest-interactions. In this sense, molecular modelling has been revealed as a powerful tool to gain deeper comprehension of these aspects, bringing up the possibility to understand, and even predict, the loading and release performance of these systems [59]. Actually, it was back in 1996 when Gusev et al., published a molecular model for MCM-41 mesoporous material [60], which was considered the “molecule of the month” in April of 1998 by Bristol University. Molecular modeling allows creating 3D models, for both the mesoporous matrix and the drug, which are then used for the docking calculations to predict the preferred orientation of the drug (ligand) into the mesoporous matrix (the receptor) to form a stable complex, i.e., minimal energy configuration [61]. Table 1 shows a selection of some representative antibiotics that have been loaded into pure-silica mesoporous matrices and then released in a controlled fashion by Vallet-Regí’s and others’ research groups. The results derived from molecular modeling and docking studies are also displayed (unpublished data).

To design the appropriate 3D model for the pure-silica mesoporous matrix, the different textural properties derived from N_2_ adsorption porosimetry measurements have to be determined. Among them, pore diameter, which is the release rate modulator, as above discussed (see Figure 2), is of foremost relevance. On this regard, MCM-41- and SBA-15-type mesoporous materials have been the most widely used host matrices for drug delivery proposes. Both materials display 2D-hexagonal structure with honeycomb arrangements of mesopores, but with different pore size, being 9–30 nm for SBA-15 and 2–10 nm for MCM-41. In addition, SBA-15-type structure contains interconnecting micropores; however, they were not taken into account to create the 3D model, since the drugs cannot penetrate into this small-size microporous channel. Accordingly, MCM-41 was used as the simplest and most useful model for these studies, and, taking into account the average data found in the literature, mesopore dimensions of 3.0 nm in diameter and 4.0 nm in length were fixed for the computational calculations.

The different electrostatic potentials maps of the different antibiotics included in Table 1 allowed predicting the functional group of the drug molecule interacting with the native silica matrix. In such maps, the atomic regions rich in electrons are typically represented in red color, whereas the electron-deficient regions are displayed in blue. In addition, other colors, such as green and yellow, are uniformly distributed and represent the covalent bonds or electron delocalization of π bonds.

**Figure 4:**
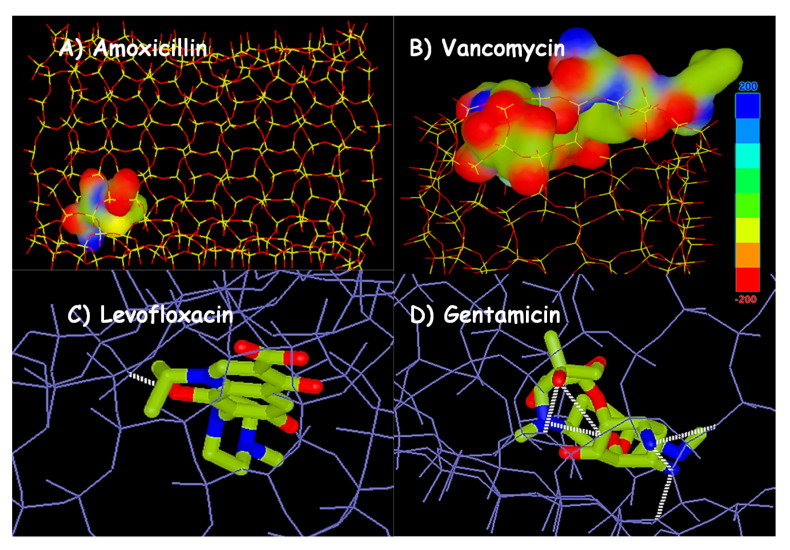
Molecular 3D models carried out with Hex 8.0 software. Electrostatic potential density maps for (**A**) amoxicillin and (**B**) vancomycin forced to penetrate into a 3.0 × 4.0 nm silica mesopore. Molecular modelling interactions studies where pure silica matrix model was used as receptor and (**C**) levofloxacin and (**D**) gentamicin as ligands.

Figure 4 shows representative examples of the application of these 3D molecular models in different MCM-41-antibiotic release systems investigated by our research group. If we observe the electrostatic potential density maps for amoxicillin (Figure 4A) and vancomycin (Figure 4B), both antibiotics exhibit regions of negative charges, and others of positive charge. In this case, the host–guest electrostatic attracting interactions between the negatively-charged silica surface and the positive regions in the antibiotic will govern drug loading and release behaviors. Such interactions are relatively weak and the magnitude of their extension depends on the size of the antibiotic. Thus, amoxicillin is small enough to penetrate into the mesoporous channels and orientates to procure interaction of its positive charged regions with the negatively charged silica surface, therefore reducing the repulsive forces between electron-rich areas (Figure 4A). On the contrary, vancomycin is too large to penetrate into the mesopore channel; therefore, it remains located on the outside of the channels, i.e., at the pore entrance, with the low electron density regions orientated to procure interaction with the negatively charged surface of the silica, as shown in Figure 4B. This predictive study agrees with the experimental results derived from loading and release assays results reported by Doadrio and coworkers [59,63].

On the other hand, hydrogen bonding host–guest interactions can be also established. These forces are stronger than electrostatic ones, and therefore they significantly influence the antibiotic release rate from pure-silica mesoporous material. Thus, the greatest the number of hydrogen bonds in the complex the slowest the release rate, as demonstrated by Doadrio et al. [74]. As representative examples, Figure 4C,D illustrate the 3D molecular models representing the hydrogen bonding that can be stablished in the MCM-41-antibiotic complexes for levofloxacin and gentamicin, respectively.

However, to fully understand the release behavior of the different antibiotics, their solubility in the aqueous medium mimicking physiological conditions cannot be overruled. Thus, the higher the solubility, the faster the release rate, as previously reported [74]. The XlogP3 values, which are closely related to the solubility, for the different investigated antibiotics are displayed in Table 1. The smaller the XlogP3 value, the higher the solubility, and therefore the faster the release rate and the greater the amount of released drug. Thus, such antibiotics showing negative XlogP3 values account for the optimal solubility in the aqueous medium.

In summary, the two parameters that mostly influence antibiotic release kinetic profiles from pure silica mesoporous matrices are the possibilities for hydrogen bonding formation and the drug solubility. For instance, experimental studies carried out by Aguilar-Colomer et al. [53] using MSNs as levofloxacin and gentamicin delivery nanodevices revealed than gentamicin, which is much more soluble in water (XlogP3 = −4.1) than levofloxacin (XlogP3 = −0.4), is more quickly released despite exhibiting a higher number of hydrogen bonds (6 hydrogen bonds) compared to the unique H-bond in the case of levofloxacin (Figure 4C,D, Table 1) [53].

## 3. A New Era of the Nanoantibiotics

**Figure 5:**
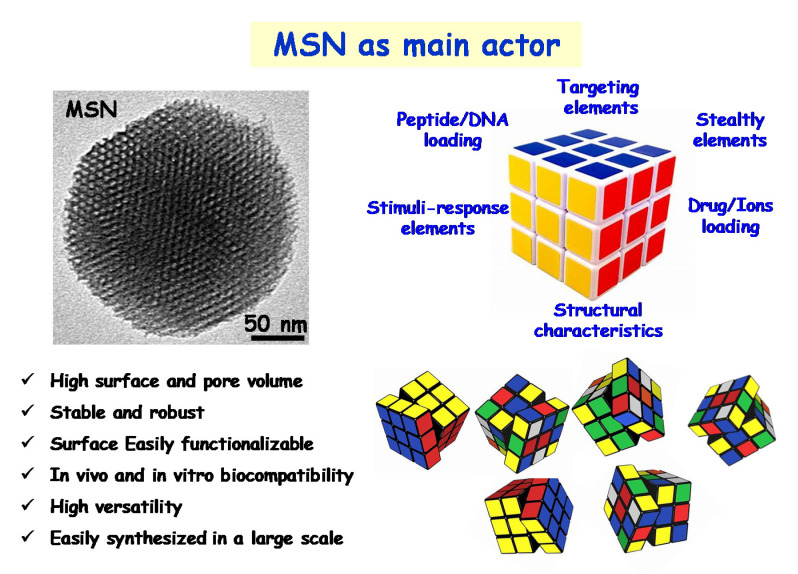
Role of MSN as one of the main actors in the design and development of a nanoantibiotic for infection treatment. Left: TEM image of a MSN and its main features as an ideal nanocarrier of antimicrobial agents. Right: illustrative simile between a MSN and a Rubik’s cube to emphasize the different facets that can be adapted to design nanoantibiotics for future personalized therapies.

In the last decade, the concept of nanoantibiotic as delivery-carrier is beginning to emerge as a very powerful therapy in the field of bacterial infection [22]. A drug delivered through nanoparticulate forms can release and specifically connect to cellular and intracellular targets, provoking much greater antimicrobial efficiency compared to the antibiotic isolated [75]. Moreover, it is a fundamental tool in the design of localised therapies, delivering large quantities of antimicrobials to the site of infection, achieving more effective therapies with lower doses, and eliminating many of the side effects. Among the different nanocarriers that can be used, the MSNs nanoparticles have been the focus of development in professor Vallet-Regí’s group [23,24,25,31,32,43,76,77]. The main strengths of MSNs as nanocarriers are their high biocompatibility and chemical stability as well as their high loading capacity, thanks to the characteristic pore lattice, and their easy functionalisation due to the presence of silanol groups. Moreover, these nanoparticles can be easily synthesized on a large scale showing a wide variety of morphologies, pore sizes, pore-lattices and surface functionalities using different strategies, thus demonstrating the great versatility of these nanosystems [31,32,42,44,47,78,79]. In this sense, MSNs can be compared to a RUBICK’s cube in which each face represents one aspect to be modified in these nanosystems, such as targeting, stealthy, peptide/protein loading, stimuli response elements, drug/ions loading and structural characteristics. Thanks to its enormous versatility and numerous positions, this “RUBICK’s cube” will give rise to multiple nanosystems to treat of different pathologies, including the bacterial infection (Figure 5). This section aims to give an overview of the main advances of these nanoparticles in the treatment of infection that have been made in professor Vallet-Regí’s group. First, it will show how MSN nanosystems are ideal nanocarriers of different antibiotics and their direct effect on the biofilm. Second, their targeting capacity towards the bacteria and the biofilm will be discussed. Third, their capacity to host different antimicrobial agents in the same nanosystem and, finally, the design of MSN-based stimuli-responsive nanosystems, will be tackled.

### 3.1. Antimicrobial Doses as Key Factor for Custom-Made Therapies

**Figure 6:**
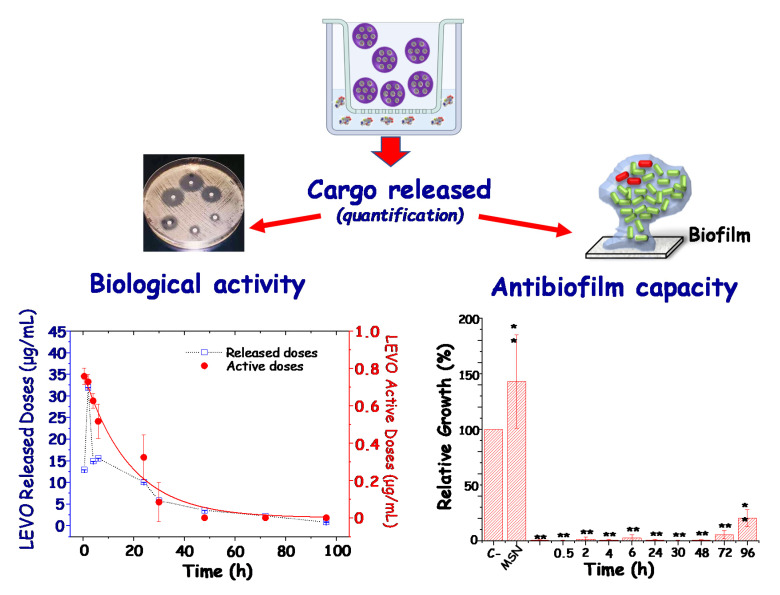
Top: Schematic depiction of the experimental design to carry out in vial drug delivery assays using antibiotic-loaded pure-silica MSNs, and subsequent quantification of cargo released doses, biological activity and antibiofilm capacity evaluations. Left graph: levofloxacin (LEVO) active doses against *S. aureus* bacteria (red curve) and LEVO released doses (blue curve) at different tested times. Right graph: In vitro antibiofilm activity of LEVO released doses in mature *S. aureus* biofilms. Adapted with permission from Aguilar et al. [53], Micropor. Mesopor. Mat., published by Elsevier, 2021.

A great number of studies have proposed multifunctional MSNs as release systems of antimicrobials for infection therapy [2,26,29,30,40,77]. In general, the antibacterial and/or antibiofilm effect of the nanosystems has been investigated as a whole, i.e., evaluating the combined effect of the different elements in the nanoplatform. Nonetheless, poor attention has been paid to the sole evaluation of the effect of the antibiotic cargoes released from MSNs. In this regard, our research group has recently reported an interesting investigation to systematically and quantitatively evaluate the active doses released from different types of antibiotic-loaded MSNs [53]. In this sense, the biological active curves together with the impact of the active antibiotic cargo on Gram-positive (*Staphylococcus aureus*) and Gram-negative (*Escherichia coli*) bacterial biofilms. In Figure 6, a schematic depiction of the experimental design to carry out this type of study is shown. In such experiments, the doses of the different antibiotic cargoes released from MSNs “in vial”, i.e., in an acellular physiological solution, as a function of time, are collected. Later, the released doses were quantified by using appropriate spectroscopic techniques; their biological activity and antibiofilm capacity was evaluated. Left graph in Figure 6 displays the active doses of levofloxacin, chosen as an illustrative example, at different times, against *S. aureus* bacteria (red curve). In addition, levofloxacin released doses during the in vial experiments are also shown (blue curve). Levofloxacin has a strong interaction with silica surface at physiological pH through hydrogen bonds with silanol groups, being that this is association responsible for the in vial release behavior [53]. As it can be observed, both active and release doses patterns are similar, but the antibiotic released doses are ca. 10-fold higher than the active doses. This discrepancy is ascribed to a partial loosening of the antimicrobial activity of the antibiotic drug. Right graph in Figure 6 shows the results derived from the evaluation of the antibiofilm capacity in *S. aureus* bacteria. It has been shown that all the released doses significantly reduced the biofilm. This antibiofilm effect is maintained over time, reducing the biofilm above 99% during the first 48 h, and achieving a reduction of ca. 77% at 96 h. This is an essential study to demonstrate that MSNs are excellent nanocarriers to load and release antibiotics of diverse families, preserving their antimicrobial activity. Thus, MSNs constitute ideal nanoplatforms as starting point towards the design of advanced nanomedicines for the management of bone infection in future personalized therapies.

### 3.2. MSNs for Targeted Delivery of Antimicrobials

The use of targeted nanoparticles represents effective new alternatives for the management of bone infection facing the two major current problems associated with infectious diseases, antimicrobial bacterial resistance and biofilm formation. Hence, the design of MSNs as antimicrobial nanocarriers against bacterial infection implies targeting strategies that lead to an enhanced efficiency of antimicrobials due to the specific interaction of the MSNs with bacteria or biofilm. In this sense, decoration of the outermost surface of MSNs with targeting ligands that produce selective accumulation in the bacteria wall or the biofilm are the approaches of foremost relevance. By using these targeting strategies, the action of antimicrobials is combined with another mechanism due to the nanocarrier itself, such as destabilisation of the bacteria wall or promotion of biofilm penetrability, therefore enhancing their efficacy. The aim is to increase the selectivity and efficiency of antimicrobials, reducing doses and frequency of the administration, therefore preventing undesirable side effects associated with unspecific drug delivery.

Just as MSNs have been successfully targeted as nanocarriers of anti-tumour drugs in the case of cancer [76,80], this concept also applies to bacterial infection. The wealth of knowledge and skills acquired in nanotechnology from basic research on smart MSN-based nanosystems for cancer therapy and diagnosis is facilitating the progress against infection by adapting to the particular characteristics of bacterial infection [29,38,40]. Active targeting provides nanosystems of specificity to the site of infection, being relevant in the case of intracellular infections, where bacteria overcome the host immune system by surviving in human cells. In this sense, MSNs have already been used as nanocarriers of anti-tuberculosis drugs [81,82], silver [83] or antimicrobial peptides [84] as antimycobacterial agents against intracellular *Mycobacterium tuberculosis*, the pathogen responsible for tuberculosis. This section describes recent advances in the pursuit of MSNs at the two main targets concerning infection management, the bacterium and the biofilm.

#### 3.2.1. Targeting Bacteria

Bacteria targeting strategies involve floating or planktonic bacteria, i.e., isolated free-living bacteria. The presence of a cell wall is the main difference between bacterial and human cells. The bacterial cell wall is a protective layer mainly consisting of peptidoglycan and other glycolipids exclusive of bacteria, which plays an essential role in bacteria growth. These distinctive elements become excellent targets for planktonic bacteria. Therefore, it is feasible to discriminate between bacteria and human host cells, by attaching the appropriate targeting moiety in the surface of the nanosystems. In addition, it is also possible to distinguish between Gram-positive and Gram-negative bacteria, attending to the different structure of their bacterial cell wall.

Gram-positive bacteria possess a cytoplasmic membrane covered by a rigid and thick layer of peptidoglycans comprising carbohydrate polymers cross-linked through peptide residues [85]. On the other hand, Gram-negative bacteria possess a triple protective layer, involving a cytoplasmic membrane and a thinner, rigid peptidoglycan layer with shorter cross-links surrounded by a hydrophobic lipid bilayer consisting of lipopolysaccharides (LPS). This lipid layer presented on the surface forms a barrier that is responsible of the great resistance of Gram-negative bacteria to several antimicrobial agents [86].

Different approaches have exploited the “ligand-receptor binding” concept, searching for highly specific targeting nanosystems, using ligands that specifically bind surface molecules or receptors overexpressed in bacteria cell walls to decorate the outermost surface of MSNs. Some of the ligands include antibodies [87,88], aptamers [89], peptides [90,91], carbohydrates [92,93] and small molecules, such as amino acids [94], vitamins [95] and certain antibiotics [96].

In addition to targeting specific components of the bacterial membrane, different adsorption pathways for the nanoparticles can also be exploited [97]. For instance, Malmsten and co-workers have investigated the lipid membrane interactions of virus-like mesoporous nanoparticles, characterized by a biomimetic “spiky” external surface. The findings demonstrate that topography influences the interaction of nanoparticles with bacteria-mimicking lipid bilayers, as well as with bacteria, producing membrane binding and destabilization. These virus-like mesoporous nanoparticles that present spikes on the external surface have been tested as carriers for the antimicrobial peptide LL-37 against *E. coli* bacteria [98]. Following this approach, Ag nanocubes with biomimetic virus-like mesoporous silica coating loaded with gentamicin are capable of effectively adsorbing on the cell wall of both *E. coli* and *MRSA*. This core–shell nanostructure can be efficiently adsorbed on the rigid cell wall due to the virus-like surface, pulling through the low cell wall adhesion capability of typical antibacterial Ag nanoparticles [99].

A step forward in the design of MSNs with a biomimetic outer shell with a bacteria targeting capability was achieved by using outer membrane vesicles (OMVs) isolated from *E. coli* as coating. As bacterial vesicles preferentially enter the same type of bacteria, owing to their similar membrane structures, MSNs loaded with rifampicin and coated with these OMVs preferentially internalize by the same type of bacteria, resulting in an enhanced antibacterial efficacy [73].

Furthermore, electrostatic attractive interactions between negative charges in the outer bacterial membrane and positively charged nanoparticles can lead to the accumulation of the latter stacked to the bacteria wall, disturbing metabolic processes or causing perforation and even membrane leakage [100,101]. Several studies have evidenced that the presence of positive charges on the surface of NPs favours internalization in both Gram-positive and Gram-negative bacteria [102]. Hence, the use of antibiotic-loaded MSNs as nanovehicles with the ability to penetrate the bacterial wall is expected to increase the antimicrobial effectiveness.

For instance, polyamine functionalized MSNs prompt cell membrane disruption in Gram-positive *Listeria monocytogene*, increasing 100-fold their antimicrobial power compared to the free polyamines [103]. By using cationic polymers such as poly-L-lysine for capping MSNs, the enhancement of antimicrobials toxicity to Gram-negative bacteria is due to the bacterial wall damage induced by positively-charged lysine residues, which allows the entrapped cargo to gain access into the bacteria [104,105].

**Figure 7:**
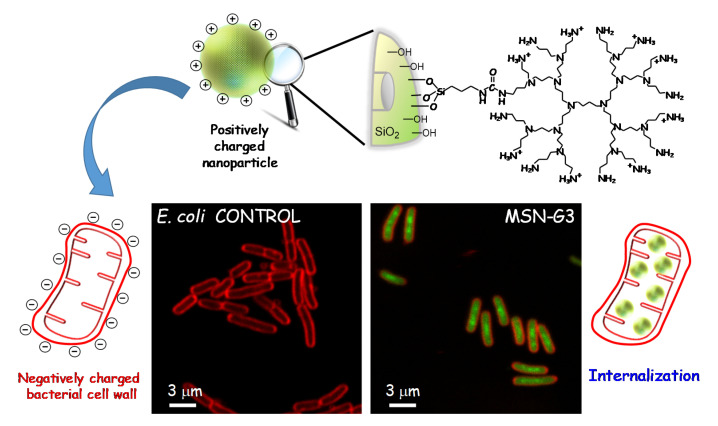
Schematic illustration showing MSNs nanosystem externally decorated with the poly(propyleneimine) dendrimer of third generation (G3) via covalent functionalization and its interaction with Gram-negative *E. coli* bacteria. The attractive forces between positive charges of the dendrimer on the surface of MSNs and negative charges on bacterial cell wall prompt cell membrane disruption and internalization of the nanosystem. Confocal microscopy images show *E. coli* control culture (left) and bacteria after exposure to 10 μg/mL of the MSN-G3 nanosystem during an incubation time of 90 min (right). The *E. coli* cell membrane was stained in red and the MSN materials have previously been fluorescently tagged in green. Adapted with permission from González et al. [55], Acta Biomater., published by Elsevier, 2018.

Examples of this kind of nanosystems developed in the Vallet-Regí’s group comprise MSNs acting as nanocarriers of levofloxacin (LEVO) as antimicrobial agent localized inside the mesopores. These “nanoantibiotics” were externally functionalized with N-(2-aminoethyl)-3-aminopropyltrimethoxysilane [56] or a polycationic poly(propyleneimine) dendrimer of third generation (G3) as targeting agents [55]. The polyamine dendrimer was covalently grafted to the outer surface of LEVO-loaded MSNs and, after physicochemical characterization, the release kinetics of loaded LEVO and the antimicrobial efficacy of each released dosage were evaluated, displaying that the antibiotic was released in a sustained fashion at effective bactericidal dosages. Moreover, internalization studies of the MSNs decorated with polycationic G3 dendrimer in *E. coli* bacteria showed a high penetrability throughout the Gram-negative bacterial membranes (see Figure 7). The high density of positive charges and flexibility on the surface of G3-MSNs produce attractive electrostatic interactions with the negatively charged bacterial walls, which triggers membrane permeabilization, and thus favours the nanosystem internalization. These studies also demonstrate that the combination of the cell wall disruption capability of G3 dendrimer and the bactericide effect of LEVO into a unique MSNs-based nanosystem has a synergistic antimicrobial effect on Gram-negative bacterial biofilm.

#### 3.2.2. Targeting Biofilm

Another challenge that society faces against bacterial infection is the ability of bacteria to form biofilms, which hinders any conventional treatment for chronic infections and has serious socio-economic implications.

Biofilms are complex bacterial communities embedded in a protective exopolysaccharide (EPS) matrix. This self-produced matrix is composed of extracellular DNA, polysaccharides, proteins, glycolipids and other ionic molecules [9]. The EPS matrix protects the bacteria from hostile environmental conditions and reduces the efficacy of antibiotics by up to 100-fold compared to planktonic cells [106]. The formation of biofilms is a multistep process in which planktonic bacteria firstly adhere to a surface. Subsequently, the bacteria multiply to form microcolonies which develop into well-defined three-dimensional structures and which eventually produce the EPS coating around them. At times, the biofilm matrix breaks and free bacteria disperse, leading to a spread in infection [107].

Therefore, the nanotechnological approaches are different when working with bacteria associated in communities forming biofilms instead of the same kind of bacteria in planktonic state. Targeting bacterial biofilms with nanocarriers capable of overcoming the barrier of the mucopolysaccharide matrix of the biofilm and releasing its loaded-antibiotic within this matrix would be highly desirable. The emerging research field regarding MSNs able to disrupt the EPS, penetrate bacterial biofilm and release the antimicrobial cargo, constitutes a promising alternative to eradicate bacterial biofilms.

Recent approaches focus on MSNs for the delivery of antibiofilm agents such as certain enzymes able to reduce EPS cohesiveness and disperse the biofilm biomass, for example lysozyme [108] or DNase I [109]. Moreover, taking into account that the EPS components typically exhibit negative charges, the nanoparticle-biofilm interactions can be increased by tailoring the surface charge of the MSNs. For instance, positively charged vancomycin-loaded MSNs were more efficiently localized to the surface of biofilm cells and were more active in reducing biofilm cell viability than MSNs having more vancomycin loaded but negatively charged [110].

In this sense, professor Vallet-Regí’s group reported the design of antibiotic nanocarriers able to penetrate bacterial biofilm using positively charged moieties as the targeting agents on the external surface of the MSNs. Amine functionalization of the MSNs with N-(2-aminoethyl)-3-aminopropyltrimethoxy-silane provides positive charges, improving the affinity of the LEVO loaded nanosystem towards the negatively charged bacteria wall and biofilm. Physicochemical characterization of the nanosystem, in vial LEVO release profiles and the in vitro antimicrobial effectiveness of the different released doses were investigated. The efficacy of this nanoantibiotic was evaluated against a *S. aureus* biofilm, showing its near-total destruction due to the high penetration ability of the developed nanosystem. Biofilm eradication is achieved thanks to the synergistic combination of antibiotic and targeting agent in a unique nanoplatform [56]. High anti-biofilm efficiency against Gram-negative *E. coli* bacteria was also accomplished throughout the synergistic action of polycationic dendrimers (G3), as bacterial membrane permeabilization agents, and LEVO loaded in the mesopores of MSNs as well [55].

**Figure 8:**
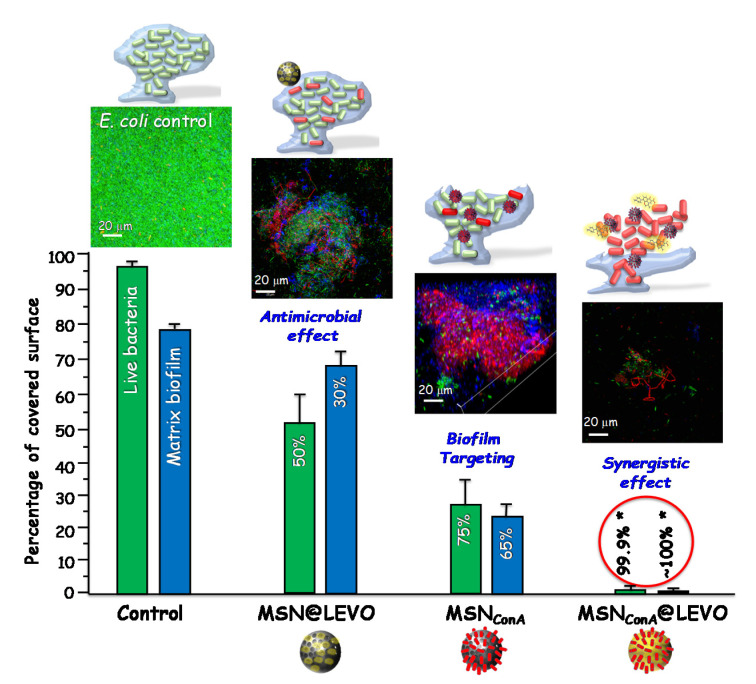
Antimicrobial activity of levofloxacin loaded and Concanavalin A functionalized MSNs against *E. coli* biofilm. The histogram represents the reduction in percentage of covered surface of live bacteria (green bars) and mucopolysaccharide matrix (blue bars), calculated from the confocal microscopy images (top). The images were obtained after exposure of a preformed biofilm to the different nanosystems during 90 min of incubation. Live bacteria are stained in green, dead bacteria in red, and the protective matrix biofilm in blue. A synergistic effect is achieved when the MSNs are loaded with levofloxacin and functionalized with ConA which acts as targeting agent for the mucopolysaccaride matrix and allows the release of the antibiotics inside the biofilm. Adapted with permission from Martínez-Carmona et al. [111], Acta Biomater., published by Elsevier, 2019.

An alternative biofilm-targeting strategy, consisting of decorating the outer surface of MSNs with molecules possessing affinity towards certain components present in the EPS, has been described by the same research group. The lectin concanavalin A (ConA) is a protein able to recognize and bind to glycan-type polysaccharides present in the biofilm EPS and, owing to this affinity, was chosen as the targeting ligand. This new nanosystem was obtained by functionalizing MSNs with carboxylic acid groups, covalently attaching ConA and loading LEVO in the mesopores. The bacterial biofilm-targeting efficacy of the nanocarrier was evaluated in *E. coli* biofilms showing that the presence of ConA in the external surface of the nanosystem promotes its internalization into the biofilm matrix in a dose dependent internalization fashion. The release of the mesopore hosted LEVO inside the biofilm is made possible thanks to the ConA-driven penetration of the nanosystem into the matrix, thus increasing the antimicrobial efficacy of the antibiotic. Hence, the synergistic combination of ConA and LEVO in the same nanoplatform lead to a complete biofilm destruction (see Figure 8) [111].

A similar approach for biofilm targeting makes use of Arabic gum as a coating of MSNs. This branched-chain complex polysaccharide is composed of 1,3-linked beta-D-galactopyranosyl monomers connected to the main chain through 1,6-linkages, whose degradation by secreted bacterial enzymes improves the retention of MSNs on the biofilm. The nanosystem demonstrated high affinity toward *E. coli* biofilm matrix, thanks to the Arabic gum coating and, loaded with two clinically relevant antibiotics such as moxifloxacin and colistin, exhibits substantial in vivo efficacy against an osteomyelitis provoked by *E. coli* [112].

### 3.3. Combined Therapies

Once the ability of these MSNs to internalise into the bacteria and the biofilm has been demonstrated, the second challenge in the fight against antimicrobial resistance consists in the design of nanosystems with enhanced antimicrobial efficacy, through the combination of different antimicrobial elements within the same nanoplatform. To this aim, different research groups have reported innovative strategies based in either the co-delivery of antibiotics [112,113,114,115], or the combination of antibiotics plus antimicrobial metal nanoparticles or ions [28].

Regarding the first approach, the most straightforward and easiest way is to simultaneously co-load different antibiotic molecules into the pores of MSNs. Thus, Gunani et al. [113] co-loaded polymyxin B and vancomycin into MSNs with high efficiency, showing high bactericidal effect against Gram-negative and Gram-positive bacteria. However, the co-encapsulation of drugs with different and occasionally opposite physico-chemical properties within a single MSN-based nanocarrier requires developing compartmentalization strategies. Along this line, Meber et al. [114] reported the synthesis of core-shell MSNs to simultaneously deliver gentamicin and rifamycin by using a multilayer construction of MSNs, following a step-by-step loading of these antibiotics. In a very recent study, Aguilera-Correa et al. [112] described an original methodology to engineer moxifloxacin-loaded MSNs coated with Arabic gum plus colistin to address *E.coli* bone infections. Significant antibacterial in vitro and in vivo efficacy was observed thanks to the Arabic gum targeting capability towards *E. coli* biofilm, the disaggregating and antibacterial effect of colistin, and a remarkable antibiofilm action due to the bactericidal activity of moxifloxacin and colistin. Another interesting methodology to co-deliver therapeutic agents of different chemical nature relies on using asymmetric MSNs with anisotropic geometry and two compartments able to load drugs of different nature in separated storage spaces. Within this context, Cheng et al. [115] reported innovative core-shell magnetic MSNs grafted to ethane-bridged mesoporous periodic organosilica with different loading properties. Gentamicin and curcumin were independently loaded into the hydrophilic and hydrophobic spaces, to provide the nanosystem of dual antibacterial and antitumor capability.

Concerning the second approach, in recent decades certain antimicrobial agents, such as some metals, metal oxides and metal salts are re-emerging in the treatment of infected prostheses. Such metals have been known for centuries for their intrinsic antibacterial properties and were used to treat bacterial and fungal infections before the discovery of penicillin by Sir Alexander Fleming. Although their medicinal utility declined with the antibiotic era, the emergence of antibiotic resistance has led to their recovery as antimicrobials. In fact, the use of some metals against bacterial infection is currently growing exponentially due to improved synthesis routes that allow the production of less toxic nanoparticles or metal oxides materials [116]. Among metal and metal oxide nanoparticles, silver nanoparticles are probably the most promising of all the inorganic nanoparticles as a treatment for bacterial infections [20]. Besides Ag, other metal nanoparticles such as Au, and metal oxide nanoparticles, such as ZnO, CuO, iron oxides (γ-Fe_2_O_3_, Fe_3_O_4_), and TiO_2_, among others, are being intensively studied for antimicrobial treatment [18,117].

However, despite the bactericidal potential of metal nanoparticles and metal ions, their use in biomedical applications is limited due to their high toxicity [118,119,120]. Therefore, a selective administration by carrying them in another vehicle would prevent systemic exposure to metallic species as well as the aggregation problems associated to uncoated metallic nanoparticles [121]. In this context, the use of MSNs as nanocarriers of silver nanoparticles has been reported for cancer [80,122] and infection treatments [83,123,124,125,126,127].

In a pioneering research project, Wang et al. [123] reported innovative core-shell nanosystems consisting in Ag nanoparticles as cores and levofloxacin-loaded MSNs as shells. This study proved that the Ag core dissolved slowly, releasing Ag^+^, which in combination with the antibiotic release, produced a synergistic antibacterial effect against drug-resistant infections both in vitro and in vivo. Very recently, and taking into account the advances achieved by María Vallet-Regí’s research group in the targeting of antibiotic-loaded nanosystems towards bacteria or biofilm using MSNs externally decorated with polyamine dendrimers (Section 3.2) [55], a totally different and groundbreaking idea was conceived, that is, using MSN-G3 nanoplatforms as vehicles for metal cations with antimicrobial properties. A great advantage of this nanoplatform is the high content in amine groups due to the dendrimer that allows for the complexation of multiactive metal cations through coordination bonds.

Therefore, a simple and versatile methodology has been recently reported by using MSNs externally decorated with G3 dendrimers for the complexation of antimicrobial metal ions (M^n+^) by soaking the MSN-G3 nanosystem into a M(NO_3_)_n_ aqueous solution [128]. This methodology allows incorporating a wide range of both antibiotics and metal ions, which permits adapting the antimicrobial cargoes to the patient clinical requirements. The selection of the cation may be done depending on clinical needs. Thus, the treatment of different pathogenic bacterial biofilms can be tailored by simply changing the loaded antibiotic attending to the clinical needs. In such work, levofloxacin, a broad spectrum antibiotic, and Zn^2+^ ion, with dual antimicrobial and osteogenic capabilities, were selected. These multicomponent nanosystems exhibited an excellent in vitro biocompatibility in MC3T3-E1 preosteoblasts cultures, and remarkable antimicrobial effect above 99% compared to the MSNs that only contained one of the antimicrobial elements. In addition, the incorporation of Zn^2+^ in the whole nanosystem promoted bone repair associated to osteolysis by stimulating bone cell differentiation. In this sense, this innovative nanosystems opens up a new scientific paradigm since the presence of metal ions may aid to prevent the emergence of antibiotic resistances, while reducing toxicity risks.

### 3.4. Stimuli-Responsive

The ideal drug nanocarrier required to fight infection must both protect the antimicrobial cargo and be able to successfully transport it to the desired cell or tissue [2,28,29,38,39,129]. When it reaches its destination, the nanocarrier must release the drug(s) in a controlled manner and in the desired concentrations. In this sense, MSNs are the perfect nanomaterial to carry out a combined therapy, since they can host in their mesopores different drugs or combinations of antibiotics that can act synergistically in the focus of the infection [2,28,29,38,39,53,77,111,128].

In order to successfully perform this function, there are several strategies to place on the pore outlets’ different organic or inorganic moieties acting as pore blockers or gatekeepers, preventing premature release of cargo [2,27,28,130,131,132]. These strategies respond to the application of various stimuli that trigger the release of the antimicrobial load at the site of infection [28,39,111]. These smart nanosystems can carry the drug content to the target cell or tissue. Once there, the presence of internal or external stimulus will trigger the drugs release in a control manner [2,28,29,32,55,111,133,134,135,136].

In the case of external stimuli an external apparatus is frequently required to trigger the release, while in the case of internal stimuli applied to nanocarriers based on MSNs no external apparatus is required [28,29,32,39,137]. However, the control over the administered dose is less than in the case of devices that use an external stimulus. In order to choose which system to use successfully, it is necessary to consider that the exact clinical application to be solved must be taken into account and the system adapted to it. Among the internal-responsive stimuli, the most used are the presence of bacteria [104], bacterial toxins [138], redox potential [139], pH [29]. Among the external-responsive stimuli, the most important are those related to chemical species [140], light [141,142,143] temperature [29] or the alternating magnetic field [29]. In addition, it is possible to combine two or more internal or external stimuli to improve the therapeutic effect of the MSN nanocarriers [2,29,144,145].

For example, among the internal-responsive stimuli, one can take advantage of the decrease in pH values that has been observed in infections, after surgery or loosening of an implant [2,29,146,147,148] to create pH stimuli-responsive nanocarriers based on MSN for detection and eradication of bacteria that only release their contents when the pH is low [2,29,149]. In these situations, the pH usually decreases from pH 7.35–7.45 in non-infected tissues to pH < 7 in bone infection and trigger some variations in the surface charge of biomaterials, in the isoelectric point and bacterial adhesion capacity, increasing the infections. Among the external-responsive stimuli, it is possible to design nanosystems based on MSN loaded with antimicrobial drugs and coated with different redox-sensitive molecules, as disulfide snap-tops [139]. This disulfide bond, and other types, can be cleaved in reducing environments and the antibiotic release is produced intracellularly, inhibiting the presence of bacteria [29].

Professor María Vallet-Regí’s research group has widespread experience in stimulus-response nanosystems based on MSNs in the treatment of several types of cancer cells, with numerous studies associated with the application of different internal and external stimuli in this area [25,28,30]. Specifically, this group has developed studies with nanosystems based on MSNs related to internal stimulus-response such as pH [150,151,152,153] or redox processes [154], and the application of several external stimuli as ultrasounds [155,156,157,158], magnetic field [159,160], photothermal therapy [161] or visible light [162]. In addition, the combination of therapeutic approaches has been proven as a complementary approach for conventional chemotherapy. In this sense, Vallet-Regi’s research group has developed nanocarriers that combined several of these internal or external stimuli [155,163,164].

**Figure 9:**
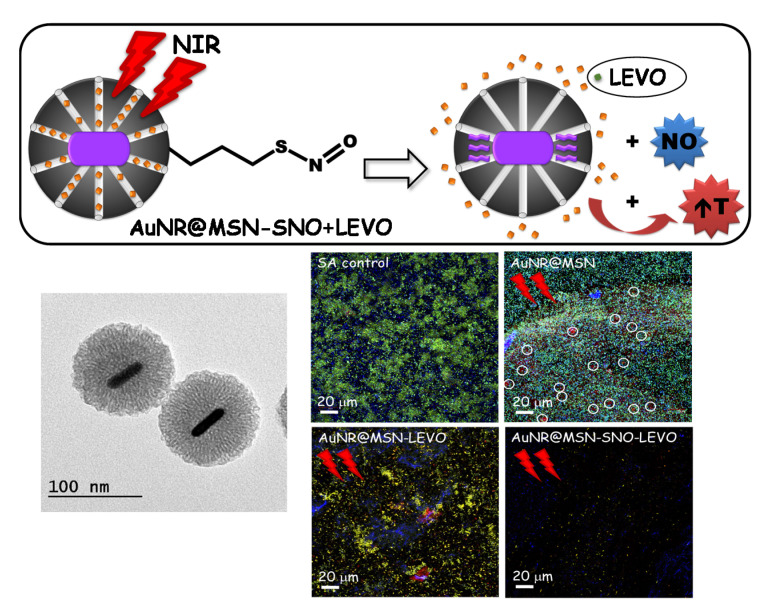
Top image: schematic representation of the nanocarrier design AuNR@MSN-SNO+LEVO and its effect on a *S. aureus* biofilm in response to near infra-red (NIR) laser treatment. Bottom left image: transmission electron microscopy micrographs of AuNRs surrounded by a mesoporous silica shell of AuNR@MSN-PEG_ext_ nanocarriers. Bottom right: confocal microscopy images of the antimicrobial action of AuNR@MSN nanocarriers onto Gram-positive *S. aureus* (SA) biofilm. These images depict the biofilm preformed without treatment (SA CONTROL), and after incubation with AuNR@MSN, AuNR@MSN+LEVO and AuNR@MSN-SNO+LEVO nanocarriers with subsequent NIR treatment. Live bacteria are stained in green, dead bacteria in red, ablation zones are marked with white circles, and the extracellular polysaccharide matrix biofilm is stained in blue. Adapted with permission from García et al. [165], Micropor. Mesopor. Mat., published by Elsevier, 2021.

The previous experience of the group in these types of stimuli-response nanosystems in the field of cancer has facilitated the possibility of developing the recent published study of María Vallet-Regí’s group, focused on the successful inhibition of biofilm through photothermal therapy with Near Infra-Red (NIR) light by gold core@shell based MSNs loaded with levofloxacin (LEVO) and nitric oxide (NO) (Figure 9) [165].

As discussed previously, bacterial biofilms can trigger chronic infections that are difficult to resolve; there are still no satisfactory alternatives to prevent and cure them [149]. In this context, different approaches for biofilm disruption were designed to destroy the protective layer and remove biofilm bacteria (DNase, proteases, cationic molecules, light-activated antimicrobial agents) [166,167,168]. María Vallet-Regí’s group has designed light-sensitive MSNs with combined photothermal (PTT) and antimicrobial properties [165]. PTT therapy has exposed a bactericidal mechanism, mostly efficient in the near NIR spectral range (650–900 nm), based on the conversion of light into localized heating and robust absorption of nanomaterials [143,169].

The development of nanosystems such as the one proposed offers a new strategy with higher efficacy, increasing the local concentration of the drug in the biofilm, and lower side actions, as it is a localized effect without causing serious injury to healthy tissues [2,29,38,53,77]. These nanocarriers acquire a great impact in the infection clinical field as they can affect the architecture of the *Staphylococcus aureus* bacterial biofilm, decreasing its growth.

On the one hand, this nanocarrier possesses photothermal therapy due to the incorporation of gold nanorods followed by the growth of a silica shell leads to a core@shell-type design (AuNR@MSN) with PTT properties. On the other hand, the LEVO is loaded into the nanocarriers’ mesoporous channels, while the nitric oxide (NO) is bonded through nitrosothiol groups (-SNO), a heat-liable linker, consequently allowing a higher NO delivery through the increase of temperature upon NIR activation [165]. Release of exogenous NO has revealed potential clinical applications in several diseases, such as cancer, cardiovascular disorders and bacterial infections [165]. Regarding infection, NO has been shown to be a crucial regulator of biofilm dispersion [170], and to trigger antibacterial action through the generation of by-products that affect oxidative and nitrosative stress [170]. In addition, it has also demonstrated its effect against biofilm dispersion when combined with conventional antibiotics [171]. The combination of these factors under a stimulus-response nanosystem produces a unique nanoassembly with potential therapeutic efficacy against *S. aureus* biofilms as can be deduced from the results obtained in this study [165]. The authors first describe the synthesis and physicochemical characterization of the nanocarriers to perform an in vitro LEVO release study with or without NIR activation and the optimization of the parameters for *S. aureus* bacterial biofilm destruction. For this purpose, preformed *S. aureus* biofilms were cultured with the different nanocarriers at a final concentration of 50 μg/mL for 90 min before first NIR laser application (808 nm, 1 W/cm^2^, 10 min). The same process was repeated after another 90 min of incubation and then all samples were incubated for 24 h at 37 °C. When light stimuli were applied, a 30% decreased of the *S. aureus* biofilm was observed with the antibiotic LEVO and its illumination with NIR irradiation displayed a biofilm reduction of 90% (Figure 9). These results denote that the combination of specific antimicrobial drug release (levofloxacin and nitric oxide) at the site of infection and PTT upon NIR irradiation disrupt the integrity of bacterial biofilms, improving the therapeutic efficiency of alternative treatments [165]. At present, Professor Vallet-Regí’s research group continues to explore more stimulus-response nanosystems to fight infection.

## 4. Conclusions

The discovery of antibiotics was one of the major breakthroughs foe humanity in the management of bacterial infections. Nonetheless, nowadays we are facing a new post-antibiotic era since bacteria have developed defense mechanisms that make them highly resistant to the existing antibiotics. This challenging issue has motivated the scientific community to develop new alternative treatments to fight infection. Nanotechnology has entered this scenario providing nanomaterials as nanocarriers of different antimicrobial agents, denoted as nanoantibiotics. Among nanocarriers, mesoporous silica nanoparticles own superlative properties and constitute ideal nanoplatforms to design targeted and stimuli-response release systems of different antimicrobial agents. These multifunctional nanosystems are foreseen as advanced antimicrobial nanoformulations for clinical application in customized therapies.

## Figures and Tables

**Table 1:** Summary of selected representative antibiotics that have been hosted into different pure-silica mesoporous matrices for controlled delivery proposes. Results derived from molecular modelling and docking studies of MCM-41-antibiotic host–guest interactions, carried out using Hex 8.0 software, are also shown (unpublished data).

Antibiotic/Host Matrix/ Antibiotic Family	Docking *	Computed Properties Drug **	H-Bonds Count **
Amoxicillin MCM-41 [62] SBA-15 [63] Semisynthetic penicillin	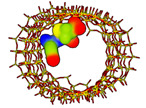 E_complex_ −227.65 kJ/mol	M_w_ = 365.4 amu XlogP3 = −2.0 Length = 10.63 Å Width = 7.90 Å	Donor drug 4 Aceptor drug 7 Complex 5
Erythromycin MCM-41 [64], MCM-41-Ti [65], SBA-15 [66], LP-Ia3d [67] Broad-spectrum macrolide	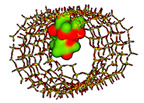 E_complex_ −315.45 kJ/mol	M_w_ = 733.9 amu XlogP3 = 2.7 Length = 14.01 Å Width = 9.70 Å	Donor drug 5 Aceptor drug 14 Complex 1
Gentamicin MCM-41 [68], SBA-15 [69] Broad-spectrum aminoglycoside	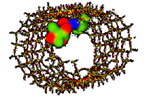 E_complex_ −256.08 kJ/mol	M_w_ = 477.6 amu XlogP3 = −4.1 Length = 14.10 Å Width = 8.90 Å	Donor drug 8 Aceptor drug 12 Complex 6
Levofloxacin MCM-41 [70], SBA-15 [71] Broad-spectrum, third-generation fluoroquinolone	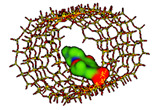 E_complex_ −241.96 kJ/mol	M_w_ = 361.4 amu XlogP3 = −0.4 Length = 14.20 Å Width = 7.50 Å	Donor drug 1 Aceptor drug 8 Complex 1
Rifampicin MCM-41 [72], MSNs [73] Semisynthetic amycolatopsis rifamycinica	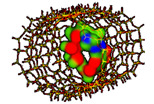 E_complex_ −349.59 kJ/mol	M_w_ = 822.9 amu XlogP3 = 4.9 Length = 14.50 Å Width = 11.50 Å	Donor drug 5 Aceptor drug 15 Complex 5
Vancomycin SBA-15 [59] Branched tricyclic glycosylated peptide	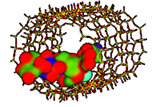 E_complex_ −486.39 kJ/mol	M_w_ = 1449.2 amu XlogP3 = −2.6 Length = 19.74 Å Width = 18.70 Å	Donor drug 19 Aceptor drug 26 Complex 4

* MCM-41 host matrix is essentially composed of negative formal charges; in drugs, red represents negative formal charges while the blue color represents positive formal charges, as shown in the electrostatic potential color scale: 
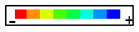
; E_complex_ is the electrostatic potential energy in the model complex, where MCM-41 was used as receptor and the antibiotic as ligand. ** XlogP3 (for the fast calculation of partition coefficient, logP), donor and acceptor drug H-bonds have been obtained from PubChem DataBank. Complex H-bonds have been obtained from Hex 8.0 docking software.

## Data Availability

Not applicable.
